# Exploring the Synergistic Effect of Simvastatin in Oral Health Applications: A Literature Review

**DOI:** 10.7759/cureus.44411

**Published:** 2023-08-30

**Authors:** Sakshi Kabra, Nilima R Thosar, Nishi S Malviya

**Affiliations:** 1 Pediatric and Preventive Dentistry, Sharard Pawar Dental College and Hospital, Datta Meghe Institute of Higher Education and Research (Deemed to be University), Wardha, IND

**Keywords:** orthodontic tooth movement, dental pulp stem cell, oral health, regenerative dentistry, statins

## Abstract

Statins are the first line of treatment for hyperlipidaemia. Along with lowering lipids, it also lowers mortality and cardiovascular risk. Statins play a major role in maintaining the homeostasis of the oral cavity via a number of different mechanisms. It includes regeneration of dentin and pulp by differentiation and increased development of mineralized tissue via the bone morphogenetic proteins (BMP)-2 Pathway. It shows effective bone health by leading to osteogenic differentiation mesenchymal stem cells, by facilitating epithelization process in wound healing, anti-inflammatory, antioxidant, antimicrobial, antiviral, and fungicidal properties. To the finest of the information we have, there have been very few comprehensive studies that have investigated the effects of statin drugs on various aspects of dental and oral health. As a result, the main objective of this review was to examine the effect of statins on oral health applications. According to the findings of our extensive review, statins have noteworthy and promising effects on several aspects of oral health, including dental pulp cells, chronic periodontitis, alveolar bone loss, orthodontic tooth movement, and so on. Nevertheless, it is concluded that local or even systemic administration of simvastatin should be regarded as an innovative, easily accessible, and safe therapeutic agent that has a significant impact on enhancing the oral health.

## Introduction and background

Statin, a hyperlipidaemic drug (3-hydroxy-3-methylglutaryl-coenzyme A). Since they have been used for so long and are the first line of treatment for hyperlipidemia, they are known to be economical and safe [1]. The statins are safe and efficient therapeutics for lowering liver cholesterol production and the production of low-density lipoprotein (LDL) cholesterol, also called bad cholesterol, which is associated with the development of cardiovascular diseases and stroke [2-5]. In addition to its effective lipid-lowering action, which lowers cardiovascular risk and death rates, statins are said to have a number of other positive effects on human health. Further, some of the pleotropic benefits of statins includes immune regulation, anti-inflammatory, enhanced endothelial function, antioxidant and anti-thrombotic qualities. Recent research reveals that statins may have positive benefits on oral and dental health via variety of mechanism [6,7].

One of the property of statin is its antimicrobial action which is most essential factor in oral health. As there is enormous pressure present on antibiotics that are now available in the healthcare system. New antimicrobials are of vital importance to combat this major public health challenge. Existing drugs with antibacterial activity that were initially approved for treatment of one clinical indication, such as lowering cholesterol levels, have the potential to accelerate the process of discovering new antibacterial agents. Based on early studies, statins, particularly simvastatin, have the potential to be modified as novel antimicrobial agents [8].

## Review

Search strategy

Data collected from various databases such as Web of Science, SCOPUS and Google Scholar using the following search terms in titles: simvastatin OR statin AND Periodontitis OR periodontal disease OR alveolar bone loss OR periodontal attachment loss OR periodontal pocket OR oral health OR oral diseases OR dental diseases OR oral cancer. The literature was searched from year 1999 to 2020, and only articles published in English were included. This review included only studies that demonstrated statistically significant results after the administration of simvastatin and if they were performed on animal or human subjects for oral health applications. Molecular in vitro studies, and simvastatin applications other than oral health were excluded from the article.

Effect of simvastatin on oral health

Simvastatin shows many positive results on oral health such as regeneration of dentin and pulp, bone health, wound healing, anti-inflammatory, antioxidant, Antimicrobial, antiviral, fungicidal properties, socket preservation, osseointegration of implants, orthodontic tooth movement or orthodontic relapse and salivary gland function [9].

Regeneration of dentin and pulp stem cells

Trauma and dental caries have the potential to harm the pulp and dentin. Liquefaction necrosis of the tooth pulp may develop as pulpal damage progresses. Pulpal necrosis stops root development and makes teeth more prone to fractures in children with immature teeth. A viable alternative for dentin regeneration has recently been identified: stem cell-based tissue engineering. The coronal portion of the dental pulp can be preserved and dentin regeneration accomplished by implanting stem cells in the pulp chamber to further restore tooth integrity [10].

The regeneration of dentin and pulp is impacted by statins. It has also been demonstrated to encourage the mineralizing phenotype in dental pulp stem cells (DPSCs). According to Okamoto et al., simvastatin-treated DPSCs show a positive impact on human odontogenic differentiation and increased the development of mineralized tissue [11]. Bone morphogenetic proteins (BMP)-2 expression is known to be accelerated by statin, which consequently improves bone growth. However, it has been found that the gene expression patterns of DPSCs are affected differently by statin and BMP-2. Particularly, statin markedly increased the expression of the dspp gene in DPSCs [12].

Statin may be useful in pulp regeneration together with dentin regeneration because it is known to enhance angiogenesis, regulate the survival of neural cells, and increase neurogenesis [13,14]. Statins also have an anti-inflammatory effect on many tissues. This might aid in healing the infected pulp tissue [15,16]. Together, these findings imply that statin would be the optimal active component of dental pulp capping agent to accelerate the synthesis of reparative dentin. The cell death seen in the cells treated with a high concentration of statin must also be taken into consideration. This fact implies that pulp tissue cells may sustain damage if statins operate at high concentrations on the cells. Therefore, prior to clinical administration, a comprehensive study is necessary to ascertain the appropriate concentration when given directly to pulp tissue or indirectly to a cavity [11].

Statins effect on bone formation

Statins have a variety of anabolic effects on bone metabolism, which have been well-documented. Statins increase the gene expression of bone morphogenic protein-2, which stimulates the differentiation of osteoblastic bone marrow stem cells (BMP-2) [17]. By preventing osteoblasts from apoptosis, they also promote bone growth. Hence, statins are known to possess a negative effect on osteoclast cells during bone remodelling, which will decrease the rate of bone resorption. Therefore, bone is continually rebuilding [12,18].

Wu et al. and Sherif et al. investigated the extraction and site preservation effects of simvastatin at 1 mg/ml in polylactic acid-co-glycolic acid (PLGA) gel and 2.5% in 1 ml chitosan gel, respectively [19,20]. Both studies came to the conclusion that simvastatin can play a key role in preserving bone height and increasing mineral density. Bone density was found to increase within a time span of four weeks, but bone height was seen to increase at the eighth week [19]. Killeen et al. added 0.5 mg simvastatin to alendronate-cyclodextrin conjugate and compared the same with alendronate-cyclodextrin conjugate alone for healing the fenestration defects associated with molar roots in rats. The study focused on the osteoconductive effect of the local application of simvastatin when given along with the systemically administered alendronate [21].

In 2011, Rutledge et al. compared simvastatin (10 mg) to porous hydroxyapatite collagen sponges that applies locally as well as injected in dog dehiscence defects [22]. Kilic et al. determined healing in closed defects, such as distraction osteogenesis, in rabbits [23]. Simvastatin was administered regionally in the amount of 2.5 mg/0.2 g gelatin and systemically in the the quantity of 10 mg. The study showed simvastatin's ability to induce bone growth at thin bone and edentulous sites [23]. Ozec et al. compared simvastatin (2.5 mg/ml) to gelatin sponges for bone density in rats with critical-sized defects [24].

The human studies showed greater homogeneity in the methodology than the animal studies [25-30]. Out of all six human studies, only one study investigated soft tissue healing potential of simvastatin. All these clinical trials aimed to determine if simvastatin intervention could benefit surgical approaches and improve clinical and radiological parameters. The dosage of simvastatin used in all studies was 1.2 mg except in the study by Gouda et al. in 2017 [29]. In this split-mouth study, 0.1 mg of simvastatin was given per 14 mg of beta-tricalcium phosphate granules (TCP) and compared with beta-TCP alone in the maxillary sinus lifting procedure. Although this study reported the outcomes of fewer patients as compared to other studies in the present review, this study is important because the patients were followed-up till nine months from baseline.

Wound healing

By preventing leukocyte adherence and extravasation into the inflamed area, statins can facilitate epithelization process and increase the rate of wound healing. This can reduce T-cell co-stimulation and the production of inflammatory cytokines. These events are important during the initial wound healing process. Statins have also increases macrophage infiltration at the healing site, which eventually promotes the growth of endothelial cells, fibroblasts and keratinocytes [30]. Following statin treatment, it has also been observed that angiogenesis is stimulated, which encourages macrophage infiltration and also causes the creation of vascular endothelial growth factor (VEGF) and re-epithelialization [30-33].

Thangamani et al. reported the anti-inflammatory and antibacterial properties of simvastatin to treat skin wounds [8]. The agent works by inhibiting various biosynthetic pathways and hampering cellular processes in bacteria, particularly affecting bacterial protein synthesis. This potential of simvastatin may be helpful in decreasing the production of methicillin-resistant Staphylococcus aureus (MRSA) toxins that downregulates the healing of septic injuries. This inhibitory action of simvastatin is peculiar to bacterial proteins, and it does not affect the synthesis process of mammalian proteins. Simvastatin was found to inhibit the growth of MRSA American-type culture collection (ATCC) 43300 strain with a minimum inhibitory concentration of 32 g/ml [8]. This property is limited to simvastatin in the statin family. However, only gram-positive bacteria can be controlled using simvastatin as the outer membrane of gram-negative bacterial prevents the entry of simvastatin.

Only one study found that simvastatin therapy was associated with disruption of normal proliferation during skin repair due to decreased VEGF protein release [34]. Simvastatin also prevents the production of Staphylococcus aureus toxin and reduces the formation of pre-formed biofilms; it can also reduce bacterial content in a mouse model of MRSA skin infection and inhibit the synthesis of bacterial protein and toxin. Simvastatin reduces inflammatory cytokines induced by MRSA skin infection and has a synergistic effect with other drugs.

Statins promotes alveolar bone formation

Tooth extraction is a transient periodontal trauma, but it causes significant alveolar bone resorption in the weeks that follow. Local application of statins in the extraction sockets can reduce the alveolar ridge resorption post-extraction [35]. Simvastatin showed alveolar bone regeneration in rat model of periodontitis. The micro-computed tomography images revealed 46% increase in alveolar height. Also, the periodontitis disease activity was reduced after simvastatin treatment. These findings shows the potential of simvastatin to increase osteoblast function and subsequent mineralisation of bone. The ease of simvastatin application at the socket may prove to be an efficient way in treating alveolar bone loss [36].

Substantial changes were found during clinical and radiological investigations in the investigational group of patients at 6 and 9 months from baseline after treatment with a statin when compared with the control group in these trials. The concentration of simvastatin used in the above clinical studies was 1.2 g/site along with the carrier which is substantially lower than the concentrations used in animal studies as reported by authors [27,36,37].

Effect of statins on orthodontic tooth movement or orthodontic relapse

As mentioned above, statin therapy not only facilitates bone formation but also prevents its resorption, thereby hampering orthodontic tooth movement. In a randomized clinical experiment to study the role of simvastatin on space re-opening after orthodontic space closure, the application of the gel containing simvastatin significantly reduced the space re-opening (p<0.001). Furthermore, the Gingival Index decrease was significantly larger (p=0.001) in the simvastatin group than in the control group. However, there was no significant difference in clinical attachment loss between the experimental and control groups [38,39].

Effect of statin on salivary gland function

Simvastatin has been studied for its radioprotective properties in rat models in which salivary gland dysfunction was induced by exposing to radiation. It could be a potential treatment option for radiation-induced hyposalivation, that reduces the quality of life for thousands of patients worldwide receiving irradiation for head and neck cancer. Exposure to ionizing radiation leads to production of reactive oxygen species such as hydroxyl radicals, superoxide, singlet oxygen, and hydrogen peroxide. These free radicals then cause damage to lipids, proteins, and nucleic acids, leading to cell dysfunction and death [40].

It has been demonstrated that reactive oxygen species can activate latent TGF-1. Significant amounts of TGF-1 produced from cells into the extracellular matrix may attract and retain inflammatory cells to an injury site, inhibit epithelial cell proliferation, and promote fibroblast maturation into post-mitotic fibrocytes, which raises fibrous tissue production. Simvastatin administration may prolong and decrease the amount of TGF-1 elevation, preserving the submandibular gland from radiation injury. Therefore, the intraperitoneal injection of simvastatin into the body may reduce salivation and restore salivary amylase activity [40].

Effect of statin on osseointegration of implants

Statins have been shown to deposit new bone around implants as well as assist in increasing the mineral density of bone [41-44]. However, for humans, the delivery route of simvastatin must be identified to get good outcomes. Simvastatin was injected intraperitoneally in certain studies [44], but statins were administered subcutaneously, orally, or intraosseously in other studies [44-47].

## Conclusions

According to the studies evaluated in this article, statins have a remarkably beneficial effect on dental pulp cells, alveolar bone loss, tissue healing, chronic periodontitis, orthodontic tooth movement, and subsequent relapse, osseointegration of implants, and salivary gland function in the oral cavity. Testing the effects and compatibility of simvastatin in clinics for various oral health issues is very important with a special emphasis on the effective dose and route of application. However, it is concluded that local and/or parenteral delivery of simvastatin may be considered a novel, easily available, and safe therapeutic agent for improving the oral health of patients.
